# Differences in avoidable mortality between migrants and the native Dutch in the Netherlands

**DOI:** 10.1186/1471-2458-6-78

**Published:** 2006-03-27

**Authors:** I Stirbu, AE Kunst, V Bos, JP Mackenbach

**Affiliations:** 1 Department of Public Health, Erasmus MC, University Medical Center Rotterdam, P.O. Box 1738, 3000 DR Rotterdam, The Netherlands

## Abstract

**Background:**

The quality of the healthcare system and its role in influencing mortality of migrant groups can be explored by examining ethnic variations in 'avoidable' mortality. This study investigates the association between the level of mortality from 'avoidable' causes and ethnic origin in the Netherlands and identifies social factors that contribute to this association.

**Methods:**

Data were obtained from cause of death and population registries in the period 1995–2000. We compared mortality rates for selected 'avoidable' conditions for Turkish, Moroccan, Surinamese and Antillean/Aruban groups to native Dutch.

**Results:**

We found slightly elevated risk in total 'avoidable' mortality for migrant populations (RR = 1.13). Higher risks of death among migrants were observed from almost all infectious diseases (most RR > 3.00) and several chronic conditions including asthma, diabetes and cerebro-vascular disorders (most RR > 1.70). Migrant women experienced a higher risk of death from maternity-related conditions (RR = 3.37). Surinamese and Antillean/Aruban population had a higher mortality risk (RR = 1.65 and 1.31 respectively), while Turkish and Moroccans experienced a lower risk of death (RR = 0.93 and 0.77 respectively) from all 'avoidable' conditions compared to native Dutch. Control for demographic and socioeconomic factors explained a substantial part of ethnic differences in 'avoidable' mortality.

**Conclusion:**

Compared to the native Dutch population, total 'avoidable' mortality was slightly elevated for all migrants combined. Mortality risks varied greatly by cause of death and ethnic origin. The substantial differences in mortality for a few 'avoidable' conditions suggest opportunities for quality improvement within specific areas of the healthcare system targeted to disadvantaged groups.

## Background

One of the factors described in the literature that influences mortality rates in developed countries is ethnic origin [1]. For some migrants a higher mortality is observed, while others benefit from lower mortality rates compared to native population [2]. Factors like socio-economic status, the healthy migrant effect, and lifestyle risk factors were shown to partly explain the differences in levels of mortality among migrant groups and the native population [3,4]. However, they do not explain the full variation in mortality outcomes.

Some researchers suggested that the healthcare system might influence mortality outcomes for migrant populations. Unequal access opportunities and sub-optimal quality of services were suggested in some studies to have contributed to ethnic disparities in mortality [5-7]. Learning more about these factors will enable health authorities to adjust the healthcare system in ways that would reduce ethnic inequalities in health.

The quality of the healthcare system and its contribution to ethnic differences in mortality could be explored by investigating 'avoidable' mortality levels [8-11]. A premature death is considered avoidable if effective measures exist (by applying appropriate preventive measures and treatment procedures on time) to avert the death of the patient [10]. Previous researches showed that mortality from 'avoidable' causes has significantly declined in the past decades in many countries [12-14] most likely due to the increased effectiveness of the healthcare services. However, a persistent ethnic gap has been shown for some countries [15,16]. An overview covering a broad range of conditions would allow pinpointing important potential problems in the delivery of health services to migrant populations.

About 10% of the population in the Netherlands is of non-Western origin with the largest representation of Turkish, Moroccan, Surinamese and Antillean/Aruban immigrant groups[17]. Recent studies have shown that Moroccans generally benefit from lower all cause mortality, while Turkish, Antilleans, and Surinamese have higher mortality rates compared to native Dutch[18,19]. Differences in avoidable mortality between migrant populations have not been documented. Thus, this study is the first to investigate the association between avoidable mortality and ethnic origin of the population in the Netherlands. We also analyze the role of socio-economic and demographic factors in this association and the influence of the duration of residence in the Netherlands on the risk of death from 'avoidable' conditions. Based on the results we will reflect on the extent to which ethnic inequalities in mortality in the Netherlands may be related to the specific problems in the Dutch healthcare system.

## Methods

### Data

The population studied comprised all inhabitants who legally resided in the Netherlands in the period 1995–2000. Data on death and population for the period 1995–2000 were obtained from the cause of death register and the Municipal Population Register that includes all inhabitants of the Netherlands with a legal status. The available data included information on sex, age, ethnicity, marital status, socio-economic status (estimated using mean household equivalent income of the neighborhoods [20]), region of residence, and urbanization degree.

Country of birth of the person and both parents was used to measure ethnicity, according to the definition used by Statistics Netherlands. If at least one parent was born abroad, the person was considered to be of non-Dutch origin. In mixed ethnic families, the country of birth of the mother prevailed [21]. We compared deaths rates of the four largest migrant groups residing in the Netherlands (1^st ^and 2^nd ^generation Turkish, Moroccan, Surinamese and Antillean/Aruban) to native Dutch.

### Selection of 'avoidable' conditions

Our selection of conditions considered avoidable was based on the original list of Rutstein et al further enlarged by Tobias and Jackson [15]. We concentrated on the role of the curative medical services, i.e. secondary and tertiary levels of care. Therefore, we included all conditions for which current evidence show that the death could be avoided by applying modern treatment, but we excluded conditions for which the outcome largely depends on primary prevention and for which curative medical care is able to play only a limited role to avoid death. The contribution of primary, secondary and tertiary levels of care for each disease was previously estimated by Tobias and Jackson [15]. Based on this information, we decided to exclude all types of injuries, smoking-related and alcohol-related conditions, and skin cancer. We included HIV/AIDS and suicides considering current evidence of effective treatment that avoids death and substantially prolongs life [22,23].

All causes of death were coded according to International Classification of the Diseases (ICD), ninth revision for the year 1995 and 10^th ^revision for the period 1996–2000. Although there is some variability in the codes between ICD revisions, the changes were not judged large enough to affect comparability over time.

All conditions were grouped into 5 subcategories depending on the type of medical service required: conditions with infectious origin, suicides, malignant neoplasms, other acute and chronic diseases, and conditions related to maternity and neonatal period (Table 2). Following Tobias and Jackson [15], the age limit 0–74 was chosen for the analysis for all causes of death.

### Analysis

Mortality levels in each population group were estimated using age-standardized mortality rates. The direct method was used with the four studied migrant groups combined as a standard population. We used pooled numbers from all migrant populations as a reference for age standardization because it better reflects the age structure of the migrant population in the Netherlands. The Dutch population is older with a considerable share of age groups above 70 years, where migrants are virtually absent. We analyzed total avoidable mortality by summing all selected causes of death.

The size of the difference in avoidable mortality rates between migrants and the Dutch population was calculated using Poisson regression (in Stata software, version 7). The resulting relative risks estimates were adjusted for age and for sex when both sexes were investigated together. Additional adjustment for marital status, urbanization level, and neighborhood area income was performed separately. These indicators were included because they were available in our dataset and because in previous analyses they have been shown to be related to cause-specific mortality rates.

To estimate the role of the duration of residence, an additional regression analysis was performed determining the risk of mortality for recent immigrants as compared to those that arrived to the Netherlands more than 15 years ago.

## Results

Turkish, Moroccans and Surinamese groups were about equally large in terms of person years at risk, while Antilleans/Arubans were about 3 times as small (Table 1). All migrant groups were more likely to live in more urbanized and low-income areas. Turkish and Moroccans were more often married than native Dutch.

We found total avoidable mortality for all studied migrant groups combined to be slightly elevated compared to the native Dutch population (RR = 1.15 for men and 1.10 for women, Table 2). The overall risk of mortality from infectious diseases was about two times higher for ethnic minorities compared to Dutch population. The relative risk for most infectious diseases was significantly elevated compared to the native Dutch. The excess risk for tuberculosis (RR = 5.10 for men and 12.98 for women) and hepatitis (RR about 8.00) was the highest, although, in absolute terms liver cancer and HIV were the two largest causes of deaths.

Compared to the native Dutch population, ethnic minorities experienced a lower risk of death from the majority of malignant conditions. Exceptions are cervical cancer and Hodgkin's disease among women, for which relative risks were insignificantly elevated. The relative risk of death for all 'avoidable' malignant conditions combined was significantly lower (RR around 0.60 for both men and women) in comparison with native Dutch.

For acute and chronic conditions combined, the mortality excess of migrant groups ranged between 22% for men and 67% for women. The risks of death from acute conditions (appendicitis, hernia, and ulcer) did not differ substantially from the Dutch population with exception of cholecysitis/cholelithiasis where the risk was three times higher among migrant men. Among chronic conditions, ethnic minorities experienced a significantly elevated risk of death from diabetes (RR above 3.00), and hypertensive and cerebro-vascular disorders (RR above 1.60). The excess risk of death from both asthma and epilepsy was significantly elevated among men, but did not reach significance level among women. Ischemic heart disease, on the other hand, was significantly higher among women (RR = 1.21 CI:1.06–1.37), but significantly lower among men (RR = 0.89 CI:0.82–0.96).

The overall risk of death from conditions related to maternity and neonatal period was 21% higher for migrant women compared to the native Dutch. Women of ethnic origin experienced an especially high mortality risk from maternity related conditions (RR = 3.37 CI:2.02–5.62). As compared to native Dutch children, children from migrant groups had higher mortality from neural tube defects (RR = 1.55 for girls with insignificantly higher levels for boys), but lower mortality from birth trauma and asphyxia (RR = 0.41 for girls with insignificantly lower levels for boys).

Socio-demographic factors (marital status, urbanization, and area income) contributed largely to the explanation of the excess mortality risks among migrant populations (Table 3). Adjustment for these factors explained about 50% of the total excess risk and for some causes of death fully explained the difference. This effect was primarily caused by control for area income instead of control for urbanization and marital status (results not shown). After these adjustments, relative mortality risks remained significantly elevated for some causes of death, including conditions of infectious origin (RR = 1.50), hypertension and cerebro-vascular diseases (RR = 1.46), and diabetes (RR = 2.65).

**Figure 1:**
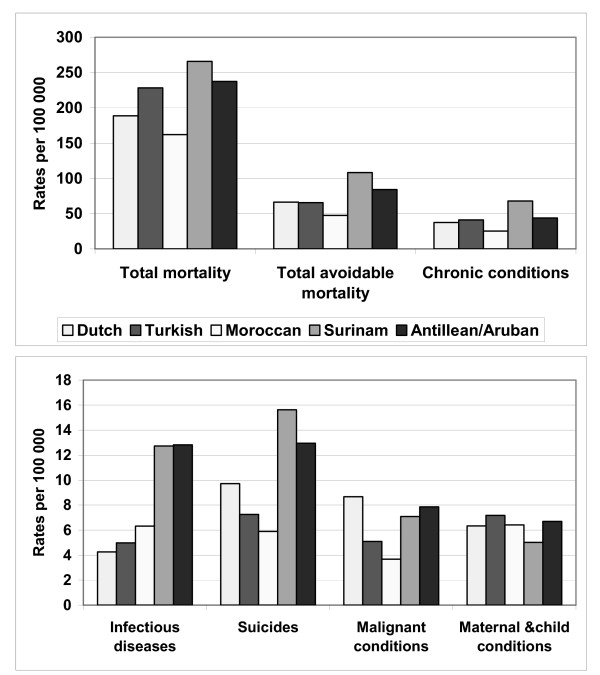
Age standardized mortality rates by condition group and ethnicity. Men and women combined.

Not all migrant groups carried an equal burden of 'avoidable' mortality risk. As shown in Fig 1 and Table 4, the Antillean/Aruban and Surinamese migrants were in a far more disadvantaged position with 23 to 50% increased risk of total avoidable mortality, while the Moroccan and Turkish population had 7 to 23% lower risk compared to the Dutch population. Adjustment for socio-economic and geographic factors explained a large portion of the excess risk of Antilleans and Surinamese populations, although some excess risk remained in the Surinamese population (RR = 1.19 CI:1.13–1.25). The lower relative risk of death for the Turkish and Moroccan population decreased even more.

All four migrant groups had a substantially elevated risk of death from infectious diseases (RRs above 1.30), but a substantially decreased mortality risk from malignant conditions (most RRs < 0.85, non significant for Antilleans/Arubans). Surinamese and Antillean/Aruban people had a significantly higher risk of death from the group of acute and chronic conditions (RR about 1.50 for men and about 2.00 for women), while Moroccan men had a significantly lower risk of death in the same group of conditions (RR = 0.68 CI:0.59–0.78). A significant excess mortality from asthma and cerebro-vascular disorders was found only among the Surinamese and Antillean/Aruban populations (RRs above 1.60). The risk of death from ischemic heart disease was increased among Surinamese migrants (RR = 1.18 for men and 1.52 for women), but decreased among Moroccan men (RR = 0.45) and insignificantly among women (RR = 0.78). A considerable excess risk of death from diabetes was found among all four migrant groups (most RRs above 2.00). Mortality risk from maternal and child conditions, on the other hand, was higher only in Turkish and Moroccan populations.

We observed a difference in risks for recent immigrants compared to those that arrived more than 15 years ago (Table 5). Recent immigrants had higher risk of death from infectious diseases and hypertension and cerebro-vascular disorders (men only) while 'older' immigrants more often died from suicides. Altogether, recent male immigrants experienced a somewhat higher risk of death from all avoidable conditions combined, while recent female immigrants had a lower risk.

## Discussion

We found total avoidable mortality to be slightly elevated for all migrant groups combined compared to the native Dutch population. Cause specific examination showed a higher risk of death among migrants from infectious and several chronic conditions and lower risk of death from malignant conditions. Ethnicity specific investigation showed that the Surinamese and Antillean groups had higher risks of death and Turkish and Moroccan groups had generally lower risks of death from 'avoidable' conditions compared to the native Dutch population. Control for demographic and socioeconomic factors explained a substantial part of ethnic differences in 'avoidable' mortality. Recent immigrants had higher risks of death from infectious diseases, but lower risk from suicides compared to those who resided longer than 15 years in the Netherlands.

Some potential limitations of the data should be considered. First, the power of the study was too limited to allow examination of all causes of death for each ethnic group separately. Second, there is a possibility of an insufficient adjustment for socioeconomic status (SES) since an ecological measure of SES based on income matched on postcode was used. It is likely that further adjustment for SES would provide additional explanation of the higher mortality for some causes in ethnic groups [24]. Third, the definition of ethnicity is based on available information on country of birth of the subject and both parents. Even though this definition is largely applied in the Netherlands, it does not take into account factors such as ethnic identity, culture, language or ancestry. As a result, it was impossible with our data to describe mortality differences within the four broad migrants groups distinguished in our study. Finally, two selection effects, 'the healthy migrant effect' and 'the unhealthy remigration effect', may have influenced the observed results. Recent studies, however, showed that they fail to explain differences in mortality between ethnic groups in Europe [19,25].

Our selection of causes of death was based on the recent work of Tobias and Jackson, and it aimed to focus on conditions that are primarily avoidable through secondary and tertiary prevention. Despite our effort to prepare a selection in a consistent way, some choices had to be made. One example is our decision to include suicide, which is based on recent evidence on the effectiveness of mental health care services to prevent a considerable part of suicides[23,26,27]. We also included ischemic heart disease (IHD) and stroke, although the contribution of non-medical factors (smoking, nutrition) to the prevention of death from IHD and stroke is large. This decision was based on the advancement in medicine that may have made the healthcare system an important determinant in shaping the patterns of IHD and stroke mortality. In absolute terms, the role of the healthcare system in preventing death from IHD and stroke is higher than for many other conditions combined. Important for the present paper is to note that any modification that may be made to our selection of causes of death, would probably not change the general conclusion that the relative level of mortality greatly varies according to 'avoidable' death, with overall levels being close to the Dutch average.

For diabetes mellitus and leukemia, our standard age interval of 0 to 74 years may be too high, as death at ages of 50 years and over becomes less 'avoidable'. The increased age-limit for diabetes and leukemia, thus, to some extent, overestimates the number of 'avoidable' deaths from diabetes and leukemia. However, it might equally overestimate the mortality risk for both the native Dutch and migrant populations. Our paper focuses on the difference in risk of death from Diabetes between native Dutch and migrant populations. We re-calculated this difference in relative risks of death from diabetes and leukemia for reduced age-limits. We found that this does not substantially change our results and still supports the conclusion that migrant populations have a significantly higher risk of death from diabetes. More specifically, the RR for diabetes in the age-group 0–49 was equal to 3.13, while in the age-group 0–74 this RR = 3.45 (Table 3). Similar results were found for leukemia (RR for age-group 0–44 = 0.90 CI: 0.68–1.21 vs. RR for age-group 0–74 = 0.81 CI: 0.60–1.15).

Additional care should be taken when interpreting the role of the healthcare system. Mortality levels are influenced by a series of factors and activities of which health care is only a part. One of the largest effects on ethnic variation in mortality may be produced by variation in incidence of the selected diseases [28]. Unfortunately, we did not have the incidence data that would be needed to perform additional adjustment for ethnic differences in incidence of infectious diseases. Furthermore, some of avoidable death could be the late consequence of inadequate care in the earlier stages of the disease before arrival to the Netherlands. Despite the problems with the validity and interpretation of the results, our overview could help identify some potential shortcomings in the healthcare system and justify further investigations in particular areas.

The decreased risk of death from ischemic heart disease among Moroccans (RR = below 0.78) might be a reflection of the healthier lifestyle that Moroccan migrants lead as compared to the native Dutch population[19]. Levels of tobacco consumption were much lower in first generation Moroccans, which is also testified by relatively low levels of lung cancer mortality. Similarly, lower levels of alcohol use and possibly a healthier traditional diet may have protected this migrant group from "western" common cardio-vascular diseases. Similar findings were reported earlier among immigrants in the Netherlands and Germany [29,30]. Given current changes in diet and smoking [31], a higher mortality may however be expected in the future and especially among second generation migrants.

Control for demographic and socioeconomic factors explained a substantial part of ethnic differences in avoidable mortality, sometimes completely abolishing the excess risk. A more comprehensive socioeconomic measure could have explained excess mortality even more substantially [24]. This indicates that socio-economic factors are important in explaining ethnic differences in mortality in the Netherlands. Similar conclusions were reached earlier by other researchers [32,33]. For a few "avoidable" causes of death, however, the situation is more complex, and adjustment for social factors only somewhat attenuated the considerably higher risks. We will discuss in more detail the possible explanations for these causes of death.

The higher risk of mortality from tuberculosis, hepatitis and chronic rheumatic heart disease among ethnic minorities in the Netherlands is likely to be the result of a higher exposure to infectious agents in the migrants' country of origin and, as a result, a higher incidence of these diseases among the migrants [34,35]. The high mortality risk can be explained, at least in part, by ethnic differences in the incidence of infectious diseases. Additional factors contributing to the higher risks of death might be substandard housing, overcrowding and poor sanitation that migrants often experience [36], partly ineffective screening programs[37], and limited access to healthcare services in the first years after migration. Although generally access in the Netherlands was found to be quite adequate [18,38], access in the first years after migration could be hampered due to financial barriers, unclear legal status and limited entitlements to healthcare, and low knowledge on the use of healthcare services. The elevated risk of death from infectious diseases among recent immigrants compared to 'older' immigrants also supports this suggestion.

The observed increased risks of death from diabetes among all four migrant groups is not a surprise and was described earlier in the Dutch literature[39]. Genetic and behavioral factors were suggested to explain the differences, among them higher low birth weight prevalence [40] and nutritional differences with higher intake of fat and carbohydrates [41]. However, some features of the present healthcare system may play an additional role by functioning less adequately for migrant groups and, thus, increasing ethnic differences in health outcomes. These include: (a) lower rate of referrals to the specialists [42] (b) somewhat less frequent use of primary healthcare facilities and poorer secondary prevention, especially among Surinamese [42]; (c) difference in the relative importance of risk factors for prediction of outcomes [43], which is not taken into account in current clinical guidelines [44]; (d) less efficient communication between providers and patients of non-Dutch origin due to cultural differences in attitudes towards health and healthcare, and illiteracy or inadequate command of Dutch language [45].

Elevated maternal mortality among migrant women is another point of concern. It may be related to fertility patterns (migrant women on the average give more often birth to children and, therefore, have a higher risk of maternal mortality per 100,000 person years), but also be related to medical services, such as reported substandard care [46], delayed prenatal care, higher frequency of unassisted births [47], and lower use of maternity home care [38]. Underreporting of maternal [48] and child [49] mortality (the last found to be associated with ethnicity) might have hindered assessment of the full extent of the ethnic gap. Elevated maternal mortality is characteristic particularly to Turkish and Moroccan groups and is not elevated among Surinamese and Antilleans. The last observation could be attributed to on average a better integration into the local Dutch society, higher local language proficiency, and more advanced education level of Surinamese and Antilleans compared to Turkish and Moroccans [50].

## Conclusion

Even though we found ethnicity to be associated with higher mortality from 'avoidable' conditions, elevated risks were confined only to specific diseases and/or separate ethnic groups. In many cases, these elevated risks were largely explained by socioeconomic and demographic factors. The role of health care system remains uncertain and is possibly weak in general. The current healthcare system in the Netherlands ensures equal financial access to healthcare services, with relatively small differences between socioeconomic groups in health care utilization[32,33]. These findings are similar to those from Sweden [51], Canada [52] and UK [7,53] where no gross ethnic inequalities in access to and utilization of the healthcare system were observed.

Nevertheless, the substantially elevated mortality levels for some 'avoidable' conditions among some migrant groups present a challenge for the healthcare system and suggest that, even though medical services may not be directly responsible, there are opportunities for quality improvement within specific areas. Areas that deserve particular attention are the control of infectious disease, care for patients with diabetes, asthma, hypertension, and maternal and neonatal care. In depth research is needed to determine more precisely the problems that migrant groups face in these areas of health care, and to develop appropriate strategies to address them.

## Competing interests

The author(s) declare that they have no competing interests.

## Authors' contributions

IS participated in the design of the study, performed the statistical analysis, and drafted the manuscript. AK conceived the study, participated in its design and coordination, and helped to bring the manuscript to its final version. VB prepared the original data files and commented on the draft paper. JM participated in the design of the study and commented on the draft paper. All authors read and approved the final manuscript.

## Pre-publication history

The pre-publication history for this paper can be accessed here:



## Figures and Tables

**Table 1:** Background characteristics by ethnicity and gender

		Dutch	Turkish	Moroccans	Surinamese	Antillean/Aruban
Person Years (*1000)	Men Women	36831 36315	904 810	780 666	810 866	264 266
Total number of deaths	Men Women	178131 108602	1967 837	1293 585	2388 3013	520 355
Total number of avoidable deaths	Men Women	62605 45687	560 272	381 211	984 698	183 145
Marital status: % single (aged 25–44)	Men Women	41.9 29.8	12.4 6.5	26.6 8.9	48.8 39.3	67.7 56.9
Duration of stay in the Netherlands: % arrived before 1980	Men Women	- -	63.3 60.2	59.1 55.1	69.9 67.8	45.6 43.5
Urbanization level: % of living in highly urbanized area	Men Women	32.8 34	74.8 74.1	77.3 77	80 80.3	66.8 69
SES: % living in low income area	Men Women	16.3 16.3	58.7 58.2	56.1 55.6	50.8 49.4	43.8 43.0

**Table 2:** Absolute numbers and relative risks of death from avoidable causes for migrant versus native Dutch population by gender

*List of conditions (ICD*^*a*^*-10 code)*	*Absolute numbers of death*^*b*^		*Relative risk*^*c *^*(95% Confidence interval)*	
	
	*Men*	*Women*	*Men*	*Women*
*Total mortality*	*6168*	*3316*	*1.18 (1.15–1.21)*	*1.12 (1.08–1.16)*
*Total avoidable mortality*	*2088*	*1318*	*1.15 (1.09–1.20)*	*1.10 (1.04–1.17)*
				
*Diseases of infectious origin*	*243*	*103*	*2.06 (1.81–2.35)*	*1.86 (1.53–2.27)*
Pneumonia & flu (J10-18)	77	42	1.25 (0.99–1.57)	1.13 (0.83–1.54)
HIV/AIDS (B20-24)	80	8	3.03 (2.39–3.85)	2.20 (1.05–4.64)
Liver cancer (C22)	46	26	2.33 (1.72–3.14)	2.49 (1.67–3.71)
Hepatitis A, B, C, D, E (B15-19)	23	8	8.54 (5.28–13.8)	7.82 (3.50–17.49)
Tuberculosis (A15-19, B90)	13	12	5.10 (2.80–9.28)	12.98 (6.85–24.61)
Chronic rheumatic heart dis. (I 00-09)	4	7	3.28 (1.17–9.19)	5.71 (2.59–12.60)
				
*Suicides *(X60-X84, Y87)	280	105	1.04 (0.92–1.17)	0.89 (0.73–1.09)
				
*Malignant diseases*	*151*	*286*	*0.63 (0.54–0.74)*	*0.56 (0.50–0.63)*
Breast cancer (C50)	0	172	-^*d*^	0.52 (0.45–0.6)
Colorectal cancer (C18-21)	89	42	0.57 (0.46–0.70)	0.40 (0.29–0.54)
Leukemia (C91-95)	54	31	0.82 (0.63–1.08)	0.75 (0.52–1.07)
Cancer cervix uteri (C53)	NA	35	NA	1.29 (0.92–1.81)
Hodgkin Disease (C81)	4	6	0.52 (0.19–1.41)	1.26 (0.55–2.89)
Cancer of testis & prostate hyperplasia (C62, N40)	4	NA	0.39 (0.15–1.06)	NA

*Acute & chronic conditions*	*1258*	*665*	*1.22 (1.15–1.29)*	*1.67 (1.55–1.81)*
Appendicitis & hernia (K35-38, K40-46, K56)	10	10	0.85 (0.45–1.60)	1.39 (0.73–2.64)
Cholecystitis & lithiasis (K80-83, K91.5)	15	3	3.04 (1.79–5.17)	0.80 (0.26–2.53)
Peptic ulcer (K25-28)	9	5	1.05 (0.54–2.03)	1.02 (0.42–2.48)
Ischemic heart disease (I20-22, I24-25)	646	253	0.89 (0.82–0.96)	1.21 (1.06–1.37)
Hypertension & cerebrovascular accident (I10-15, I61- 66, I67.4)	281	187	1.64 (1.45–1.85)	1.71 (1.48–1.98)
Diabetes (E10-14)	254	184	3.10 (2.73–3.53)	3.99 (3.43–4.65)
Epilepsy (G40-41)	32	15	1.51 (1.05–2.18)	1.15 (0.68–1.96)
Asthma (J45-46)	11	8	2.72 (1.43–5.19)	1.76 (0.85–3.64)
				
*Conditions related to maternity & neonatal period*	*156*	*159*	*0.94 (0.84–1.15)*	*1.21 (1.04–1.46)*
All Maternal deaths (O 00–99)	NA	19	NA	3.37 (2.02–5.62)
Congenital anomalies (P10-15, P20-21, P50-51, P95)	75	59	0.98 (0.77–1.25)	1.23 (0.93–1.61)
Other neonatal deaths (P08, P22, P25-26, P28, P52-P94, P96)	34	29	0.38 (0.25–0.60)	0.97 (0.66–1.43)
Neural tube defects (Q00-07)	25	35	1.12 (0.74–1.69)	1.55 (1.08–2.22)
Birth trauma & asphyxia (Q10- 23.3, Q23.8-28, Q35-84)	22	17	0.90 (0.58–1.40)	0.41 (0.2–0.84)

^*a *^International Classification of the Diseases

^*b *^For all migrant population combined

^*c *^All migrant groups combined compared to the native Dutch population, adjustment for age

^*d *^No cases

**Table 3:** Relative risks of death from groups of conditions for all migrant groups combined compared to native Dutch population. Men and women combined

	Relative risk^*a *^(95% Confidence interval)	
	
	*Adjusted for age and sex*	*Adjusted for all factors*^*b*^
*Total mortality*	*1.16 (1.13–1.18)*	*0.98 (0.96–1.00)*
*Total avoidable mortality*	*1.13 (1.09–1.17)*	*0.97 (0.94–1.01)*
		
Diseases of infectious origin	2.00 (1.79–2.23)	1.50 (1.32–1.70)
Suicides	0.99 (0.90–1.10)	0.83 (0.74–0.93)
Malignant diseases	0.58 (0.53–0.64)	0.55 (0.50–0.61)
Acute and Chronic conditions	1.36 (1.29–1.42)	1.17 (1.11–1.23)
Ischemic heart disease	0.97 (0.90–1.03)	0.81 (0.76–0.87)
Asthma	2.20 (1.36–3.56)	1.66 (0.99–2.76)
Hypertension & cerebro-vascular accident	1.67 (1.53–1.84)	1.46 (1.32–1.60)
Diabetes (age 0–74)	3.45 (3.13–3.81)	2.65 (2.38–2.94)
Diabetes (age 0–49)	3.13 (2.48–3.96)	2.54 (2.22–2.87)
Conditions related to maternity & neonatal period	1.10 (0.99–1.24)	1.04 (0.92–1.20)

^*a *^Migrant groups compared to the native Dutch population, adjustment for age

^*b *^Adjustment for age, gender, marital status, urbanization level, and area income

**Table 4:** Relative risk of death from groups of conditions for different migrant groups compared to native Dutch population

	Relative Risk^*a *^(95% Confidence interval)			
	
	Turkish	Moroccans	Surinamese	Antillean/Aruban
***Men & women***				
*Total mortality*	*1.15 (1.11–1.20)*	*0.90 (0.86–0.94)*	*1.33 (1.29–1.37)*	*1.27 (1.18–1.35)*
*Total avoidable mortality*	*0.93 (0.86–0.99)*	*0.77 (0.71–0.84)*	*1.50 (1.43–1.57)*	*1.23 (1.13–1.4)*
*Total avoidable mortality adjusted for all factors*^*b*^	*0.86 (0.81–0.93)*	*0.72 (0.66–0.78)*	*1.19 (1.13–1.25)*	*1.00 (0.90–1.11)*
				
***Men***				
*Total mortality*	*1.22 (1.16–1.27)*	*0.86 (0.81–0.90)*	*1.40 (1.34–1.45)*	*1.35 (1.24–1.47)*
*Total AM*	*0.99 (0.91–1.07)*	*0.72 (0.65–0.80)*	*1.64 (1.54–1.75)*	*1.36 (1.18–1.57)*
Conditions of infectious origin	1.28 (0.95–1.71)	1.48 (1.12–1.98)	2.89 (2.40–3.47)	3.56 (2.51–5.05)
Suicides	0.77 (0.60–0.98)	0.61 (0.46–0.83)	1.58 (1.33–1.88)	1.38 (0.99–1.93)
Malignant diseases	0.58 (0.40–0.78)	0.42 (0.29–0.60)	0.81 (0.64–1.04)	0.88 (0.53–1.46)
Acute and chronic conditions	1.08 (0.97–1.20)	0.68 (0.59–0.78)	1.79 (1.65–1.93)	1.25 (1.02–1.55)
Ischemic heart disease	1.00 (0.87–1.14)	0.45 (0.37–0.55)	1.18 (1.05–1.32)	0.80 (0.59–1.10)
Asthma	0.77 (0.11–5.57)	1.77 (0.43–7.25)	3.20 (1.16–8.79)	11.23 (4.05–31.14)
Hypertension & cerebro- vascular accidents	1.25 (0.97–1.60)	0.80 (0.58–1.10)	2.61 (2.23–3.05)	1.68 (1.07–2.64)
Diabetes	1.72 (1.27–2.32)	1.96 (1.47–2.62)	5.29 (4.48–6.25)	3.25 (2.04–5.17)
Conditions related to neonatal period	1.14 (0.90–1.44)	1.01 (0.78–1.31)	0.78 (0.54–1.11)	1.01 (0.60–1.72)
				
***Women***				
*Total mortality*	*1.04 (0.97–1.11)*	*1.00 (0.92–1.08)*	*1.23 (1.17–1.29)*	*1.16 (1.05–1.29)*
*Total AM*	*0.83 (0.73–0.93)*	*0.89 (0.78–1.02)*	*1.34 (1.24–1.44)*	*1.15 (0.98–1.36)*
Conditions of infectious origin	1.45 (0.95–2.22)	1.97 (1.27–3.04)	1.97 (1.49–2.61)	2.12 (1.23–3.67)
Suicides	0.59 (0.38–0.92)	0.56 (0.33–0.95)	1.29 (1.00–1.67)	0.98 (0.55–1.73)
Malignant diseases	0.46 (0.36–0.59)	0.44 (0.33–0.60)	0.61 (0.51–0.72)	0.84 (0.62–1.12)
Acute and chronic conditions	1.09 (0.90–1.32)	1.09 (0.86–1.39)	2.18 (1.98–2.40)	1.46 (1.14–1.88)
Ischemic heart disease	0.89 (0.66–1.19)	0.78 (0.52–1.15)	1.52 (1.30–1.77)	1.10 (0.74–1.63)
Asthma	0.77 (0.11–5.56)	0.99 (0.14–7.13)	3.35 (1.46–7.65)	-^*c*^
Hypertension & cerebro- vascular accidents	0.99 (0.67–1.46)	1.08 (0.68–1.72)	2.26 (1.90–2.71)	1.60 (1.02–2.52)
Diabetes	2.17 (1.46–3.22)	2.29 (1.42–3.70)	5.54 (4.62–6.64)	3.63 (2.28–5.78)
Conditions related to maternity & neonatal period	1.31 (1.01–1.70)	1.52 (1.19–1.96)	0.89 (0.61–1.30)	1.32 (0.78–2.23)

^*a *^Migrant groups compared to the native Dutch population, adjustment for age, adjustment for age

^*b *^Adjustment for age, gender, marital status, urbanization level, and area income

^*c *^No cases

**Table 5:** Relative risk of death from groups of conditions for recent ^*a *^migrants compared to migrants residing in the Netherlands 15 years or longer^*a *^Arrived less than 15 years ago to the Netherlands ^*b *^Recent migrants compared to migrants residing in the Netherlands 15 years or longer. Adjustment for age and gender (column men & women)

	Relative Risk ^*b *^(95% confidence interval)		
	
	*Men*	*Women*	*Men & women*
*Total mortality*	*1.08 (1.02–1.15)*	*0.94 (0.87–1.01)*	*1.02 (0.98–1.07)*
*Total avoidable mortality*	*1.13 (1.02–1.25)*	*0.88 (0.78–0.98)*	*1.01 (0.94–1.09)*
			
Conditions of infectious origin	1.64 (1.24–2.18)	1.42 (0.96–2.11)	1.62 (1.29–2.03)
Suicides	0.72 (0.57–0.93)	0.59 (0.39–0.87)	0.68 (0.55–0.84)
Malignant diseases	1.09 (0.74–1.60)	0.90 (0.71–1.15)	0.92 (0.75–1.13)
Acute and chronic conditions	1.19 (1.04–1.36)	0.84 (0.72–0.98)	1.04 (0.94–1.15)
Ischemic heart disease	1.09 (0.91–1.32)	0.90 (0.70–1.16)	1.04 (0.89–1.21)
Asthma	0.50 (0.12–2.05)	0.37 (0.07–1.92)	0.46 (0.16–1.34)
Hypertension & cerebro- vascular accidents	1.49 (1.14–1.93)	0.67 (0.49–0.91)	1.04 (0.85–1.28)
Diabetes	1.29 (0.96–1.72)	1.05 (0.78–1.41)	1.17 (0.95–1.44)
Conditions related to maternity & neonatal period	1.36 (0.56–3.30)	1.0 (0.52–1.93)	1.12 (0.65–1.91)

^*a *^Migrant groups compared to the native Dutch population, adjustment for age, adjustment for age

^*b *^Adjustment for age, gender, marital status, urbanization level, and area income

^*c *^No cases
